# Review and optimization of image classification models for edge AI applications

**DOI:** 10.1186/s40580-026-00574-w

**Published:** 2026-09-22

**Authors:** Sunghyun Kim, Hanbin Cho, Taejee Kim, Huseok Lee, Sungjae An, Sungju Ryu

**Affiliations:** 1 Department of Electronic Engineering, Sogang University, Seoul, 04107 South Korea; 2 Department of System Semiconductor Engineering, Sogang University, Seoul, 04107 South Korea

**Keywords:** Image classification, Vision transformer, Edge AI, Token merging

## Abstract

Image classification on edge devices requires models that maintain accuracy while reducing inference cost. This paper reviews the development of image classification models from convolutional neural networks to Vision Transformer models and examines their relevance to edge artificial intelligence applications. To improve Vision Transformer inference efficiency without redesigning the backbone, we propose a selective token merging method that identifies low-significance image tokens and applies token merging mainly to those tokens. Experimental results on CIFAR-10 using ViT-Tiny show that the proposed method improves the accuracy-throughput tradeoff compared with standard Token Merging. In particular, selectively merging the least significant token group with a layer-dependent schedule achieves 97.41% accuracy and 19.04 images/s, compared with 97.22% accuracy and 18.97 images/s for standard Token Merging. Additional ImageNet-1 K evaluations using ViT-Tiny, ViT-Small, and ViT-Base show that selective merging in an shallow-layer window preserves more of the baseline accuracy than standard ToMe while retaining an inference speedup. These results show that token significance can guide more accurate and efficient token reduction for edge-oriented image classification.

**Graphical abstract:**

**Figure:**
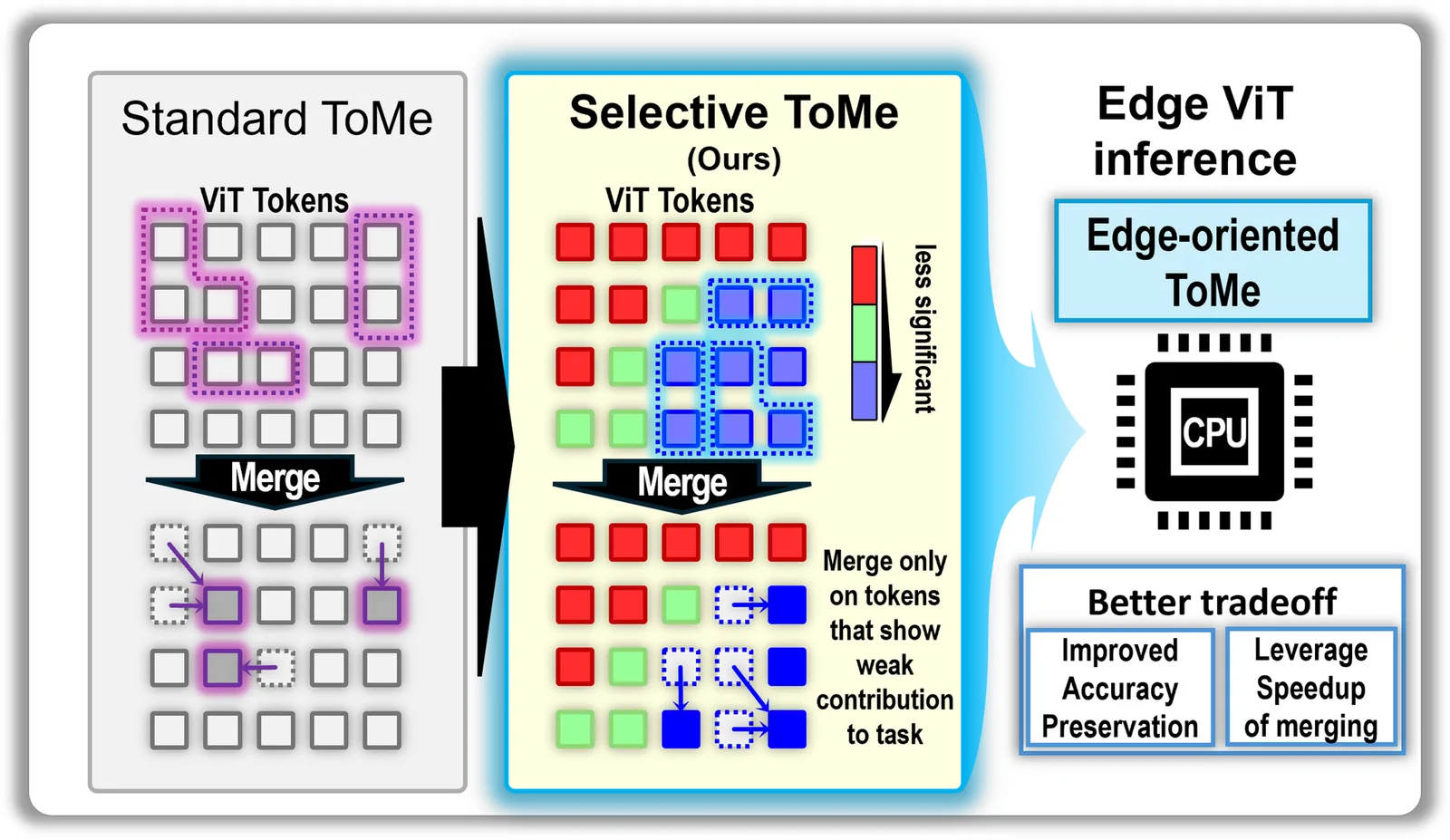

## Introduction

**Fig. 1:**
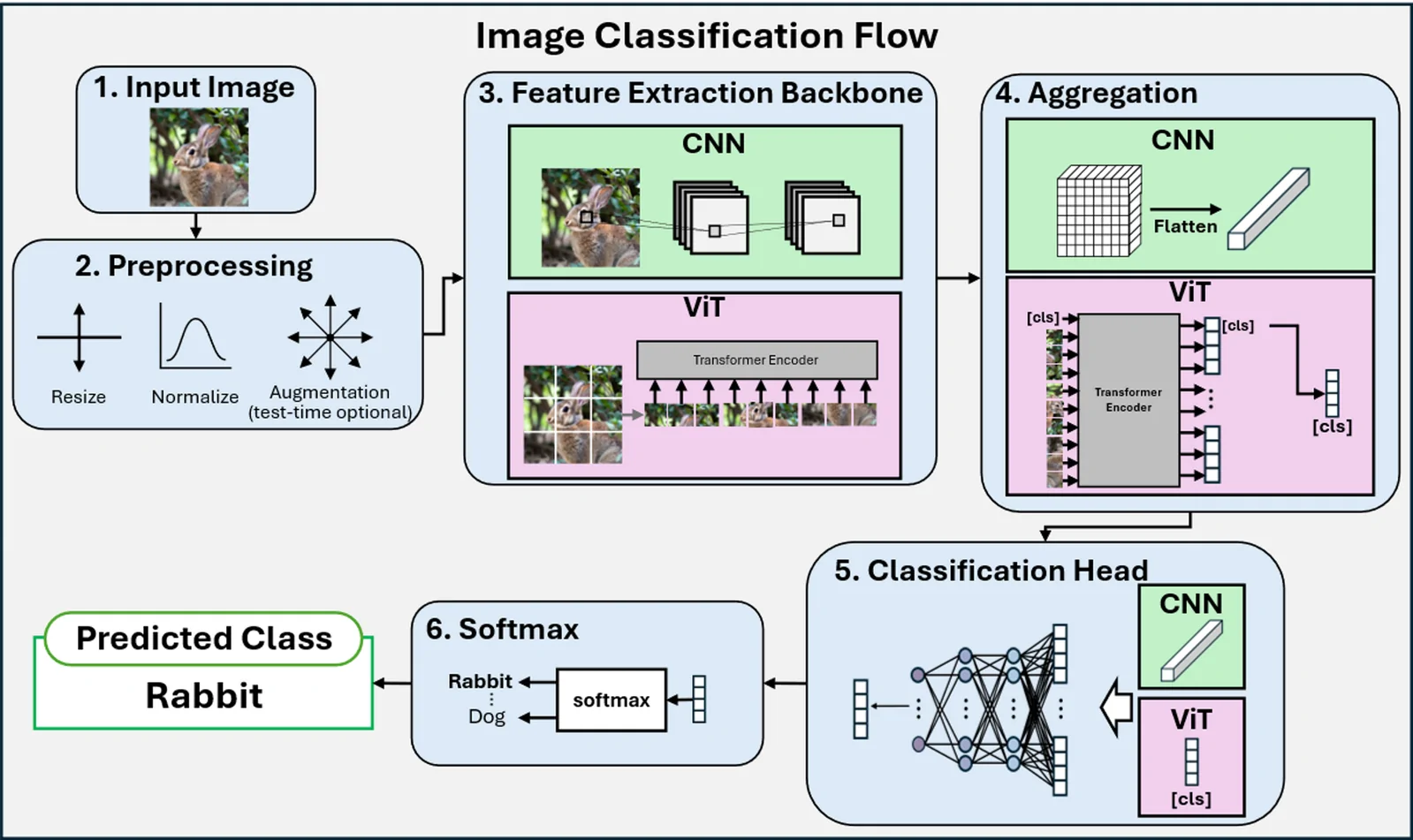
Image classification inference flow. An input image is preprocessed and passed through a feature-extraction backbone, where CNN aggregate spatial feature maps and ViT aggregates patch-token representations through a Transformer encoder. The resulting image-level representation is processed by a classification head and softmax layer to produce the predicted class

Image classification maps an input image to a semantic class label through a sequence of preprocessing, feature extraction, feature aggregation, and classification, as summarized in Fig. 1. During inference, the input image is resized, normalized, and transformed into the representation required by the backbone model. The backbone extracts visual features and aggregates spatial or token-level information into an image-level representation, which is then processed by a classification head to produce class probabilities. In edge AI applications, this pipeline must satisfy strict latency, memory, and energy constraints, making the computational cost of the feature-extraction backbone a central design concern.

Convolutional neural networks (CNNs) became the dominant model family for image classification after AlexNet and were further strengthened by deeper residual architectures such as ResNet [1, 2]. Efficient CNNs such as MobileNet then showed that architecture design could be explicitly adapted to mobile and embedded inference [3]. Vision Transformer (ViT) later reformulated image classification as patch-token sequence modeling with a Transformer encoder, shifting visual recognition from convolution-dominated backbones toward attention-based representations [4]. However, vanilla ViT remains expensive for edge deployment because full self-attention scales quadratically with the number of image tokens [4, 5].

Designing lightweight ViT variants is attractive, but preserving the vanilla ViT structure is also valuable because many strong pretrained and self-supervised models are built around standard ViT backbones [6]. Instead of replacing the model architecture, token-reduction methods can improve inference efficiency by modifying the token sequence processed by an existing ViT. Prior token-pruning and token-reorganization studies show that not all image tokens contribute equally to classification, while Token Merging (ToMe) shows that token aggregation can improve throughput without requiring full retraining [7].

In this paper, we first review representative image-classification models from CNN-based architectures to ViT-based architectures, and further examine prior efforts toward efficient edge AI inference. Based on the observation that image tokens contribute unequally to classification, we propose a selective token merging method that further optimizes ToMe for edge AI applications while keeping the vanilla ViT backbone largely intact. We then evaluate multiple selective-merging configurations and show that merging tokens with the lowest classification relevance can increase throughput while preserving accuracy better than the original ToMe.

## Review of image classification models

Image classification is one of the central tasks in computer vision. Since the ImageNet-scale success of AlexNet and later deep CNN architectures, convolutional neural networks (CNNs) have been the dominant model family for image recognition [2]. In parallel, Transformers became highly influential in natural language processing, and ViT later introduced a direct Transformer-based formulation for image classification by representing an image as a sequence of patches [5]. This section reviews representative CNN-based and ViT-based models that are relevant to the development of image classification and to edge AI deployment.

### CNN-based models

**Fig. 2:**
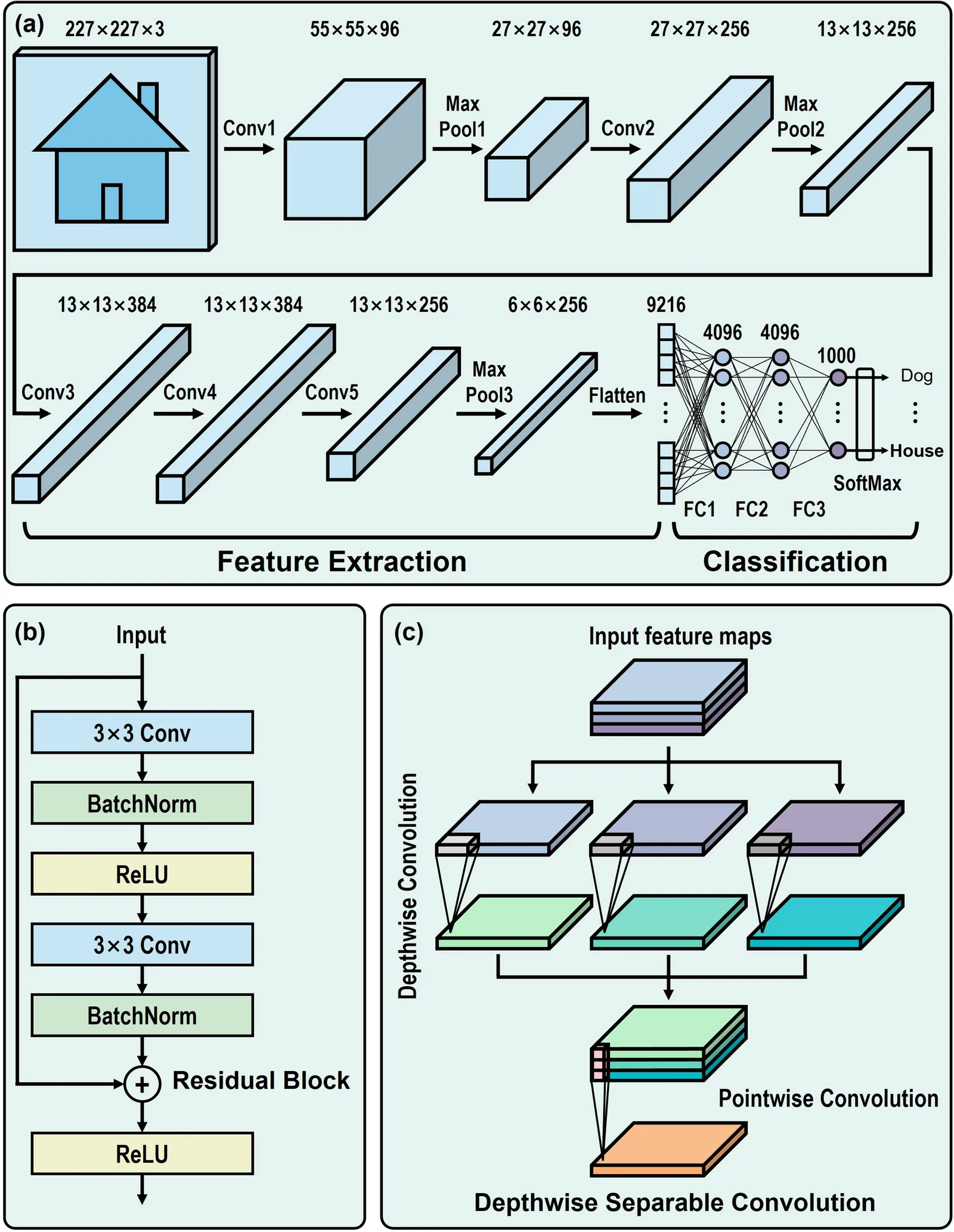
Representative CNN architectures and building blocks for image classification. **a** AlexNet-style convolutional classification pipeline with convolution, pooling, fully connected layers, and softmax classification. **b** Residual block used in ResNet, where shortcut connections support deeper CNN optimization. **c** Depthwise separable convolution used in MobileNet, factorizing standard convolution into depthwise and pointwise operations for efficient inference

**AlexNet** is commonly cited as a landmark CNN architecture in modern image classification. It demonstrated that large deep convolutional neural networks trained on ImageNet-scale data could substantially outperform previous visual recognition methods; a variant/ensemble associated with AlexNet won ILSVRC-2012 with a top-5 test error of 15.3%, compared with 26.2% for the second-best entry [2]. Architecturally, AlexNet used five convolutional layers and three fully connected layers, together with ReLU activations, max pooling, data augmentation, dropout, and GPU-based training, as illustrated in Fig. 2a. Its success was important not because each component was individually new, but because it showed that large-scale deep feature learning could be a practical and highly effective approach for image classification. After AlexNet, CNNs became the dominant model family for large-scale visual recognition.

**ResNet** further advanced CNN-based image classification by enabling the effective training of much deeper networks. As CNN depth increased, plain networks suffered from a degradation problem in which adding more layers could increase training error rather than improve performance. ResNet addressed this problem through residual learning, where stacked layers learn a residual function with respect to the input and shortcut connections pass information across layers [1], as shown in Fig. 2b. This design made it possible to train very deep architectures such as ResNet-50, ResNet-101, and ResNet-152, achieving strong ImageNet classification performance and influencing many later vision backbones. In the development of image classification models, AlexNet represents the large-scale adoption of deep CNNs, while ResNet represents the transition toward very deep, modular, and easier-to-optimize CNN architectures.

**MobileNet.** Although AlexNet and ResNet were primarily developed as accuracy-driven image-classification architectures, MobileNet was designed for mobile and embedded vision applications. MobileNetV1 built its architecture mainly from depthwise separable convolutions, which factorize a standard convolution into a depthwise spatial convolution and a pointwise 1×1 convolution [3], as shown in Fig. 2c. This factorization greatly reduces computation and parameter count relative to standard convolution. MobileNetV1 also introduced width and resolution multipliers, allowing model size and computational cost to be adjusted according to target device constraints [3]. Thus, MobileNetV1 marked an important shift from general CNN scaling toward explicitly efficient CNN design.

MobileNetV2 improved the MobileNet design through inverted residual blocks and linear bottlenecks. In contrast to conventional residual blocks, MobileNetV2 expands a low-dimensional input representation into a higher-dimensional intermediate representation, applies lightweight depthwise convolution, and then projects the result back to a low-dimensional bottleneck [8]. Shortcut connections are placed between bottleneck representations, and the final narrow projection is kept linear to reduce information loss. These changes made MobileNetV2 an important refinement of compact CNN design for efficient image classification [8].

MobileNetV3 continued this progression by combining platform-aware neural architecture search with NetAdapt-based refinement, making mobile latency an explicit design objective [9]. The MobileNet series is therefore important in a review of image classification because it shows how CNN architectures evolved from high-accuracy deep models toward lightweight, hardware-aware models suitable for edge and mobile inference.

### Vision transformer (ViT) models

**Vision transformer (ViT)** introduced a major alternative to convolution-based image classification by applying the Transformer architecture directly to image patches. In the vanilla ViT formulation, an input image is divided into fixed-size patches, each patch is linearly embedded as a token, positional embeddings are added, and the resulting token sequence is processed by a standard Transformer encoder [4]. A learnable class token is appended to the sequence and used as the image-level representation for classification. This formulation translated image classification into a sequence modeling problem, connecting computer vision to the self-attention mechanisms that had become dominant in natural language processing [5].

**Fig. 3:**
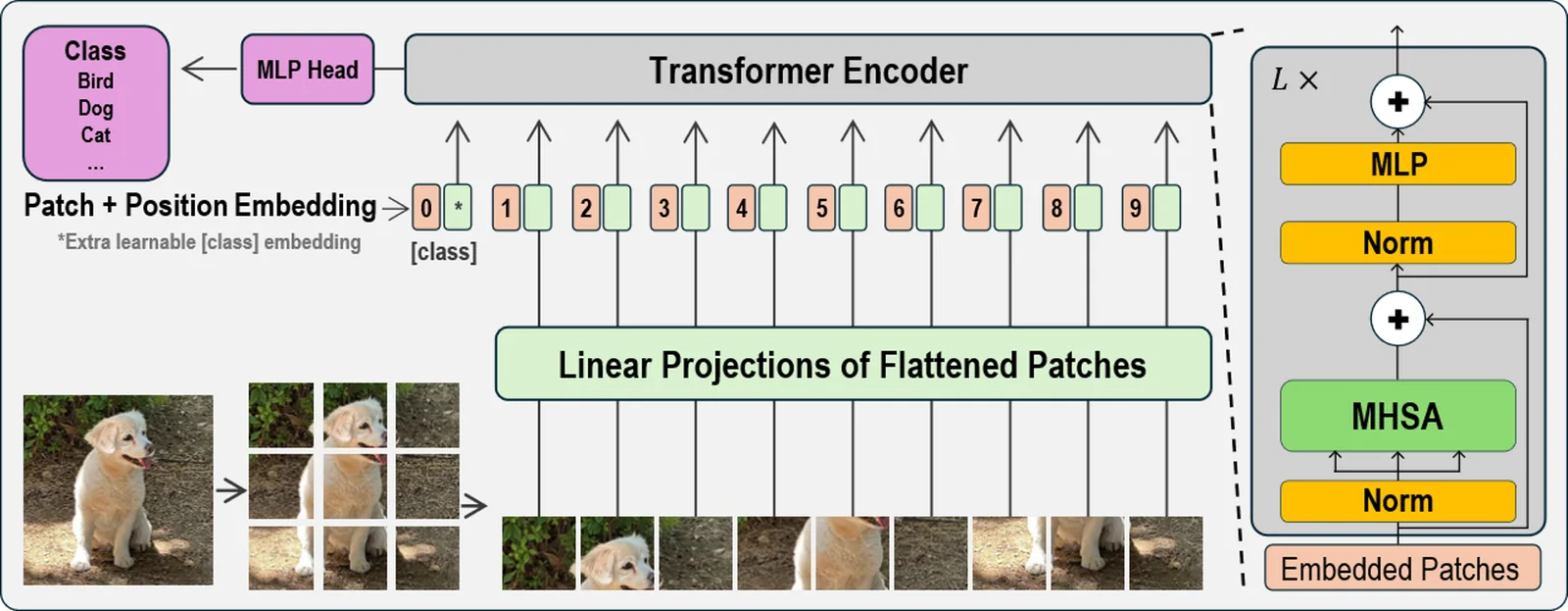
Vision Transformer architecture for image classification. An input image is divided into patches, linearly embedded with positional information, and processed with an added learnable class token through repeated Transformer encoder blocks. The final class-token representation is passed to an MLP head for classification

As shown in Fig. 3, the Transformer encoder is composed of repeated blocks containing multi-head self-attention (MHSA) and a feed-forward network (FFN), often implemented as a multilayer perceptron (MLP). MHSA projects token embeddings into queries, keys, and values, computes attention scores among tokens, and aggregates value vectors so that each token can incorporate global image context. The FFN/MLP is applied independently to each token to transform its channel representation after attention. Residual connections and layer normalization around these sublayers stabilize optimization, while the final class-token representation is passed to the classification head.

ViT attracted attention because it challenged the long-standing assumption that strong image-specific inductive biases, such as locality and translation equivariance, must be built into the model architecture. CNNs encode these biases through convolutional kernels and hierarchical feature maps. ViT, by contrast, uses only limited image-specific structure: the image is split into patches, and the Transformer encoder learns interactions among tokens through self-attention [4]. The original ViT work showed that, when pretrained on sufficiently large datasets, pure Transformer models could match or outperform strong CNN-based baselines on image recognition tasks. At the same time, this result was data-scale dependent: ViT models trained with less data were less competitive because they lacked the inductive biases built into CNNs [4].

A key limitation of vanilla ViT is its computational and memory cost, especially for edge AI applications. Let *N* denote the number of tokens, *d* the token embedding dimension, and *h* the number of attention heads. In image classification, *N* depends on image resolution and patch size, so smaller patches or higher-resolution inputs increase the attention computation and the $O\left(h{N}^{2}\right)$ attention-memory footprint [5]. In addition, projection and FFN layers add $O\left(N{d}^{2}\right)$-scale computation and memory traffic. Compared with lightweight CNNs such as MobileNet, vanilla ViT is therefore less directly suited to resource-constrained deployment without architectural or algorithmic optimization.

**Fig. 4:**
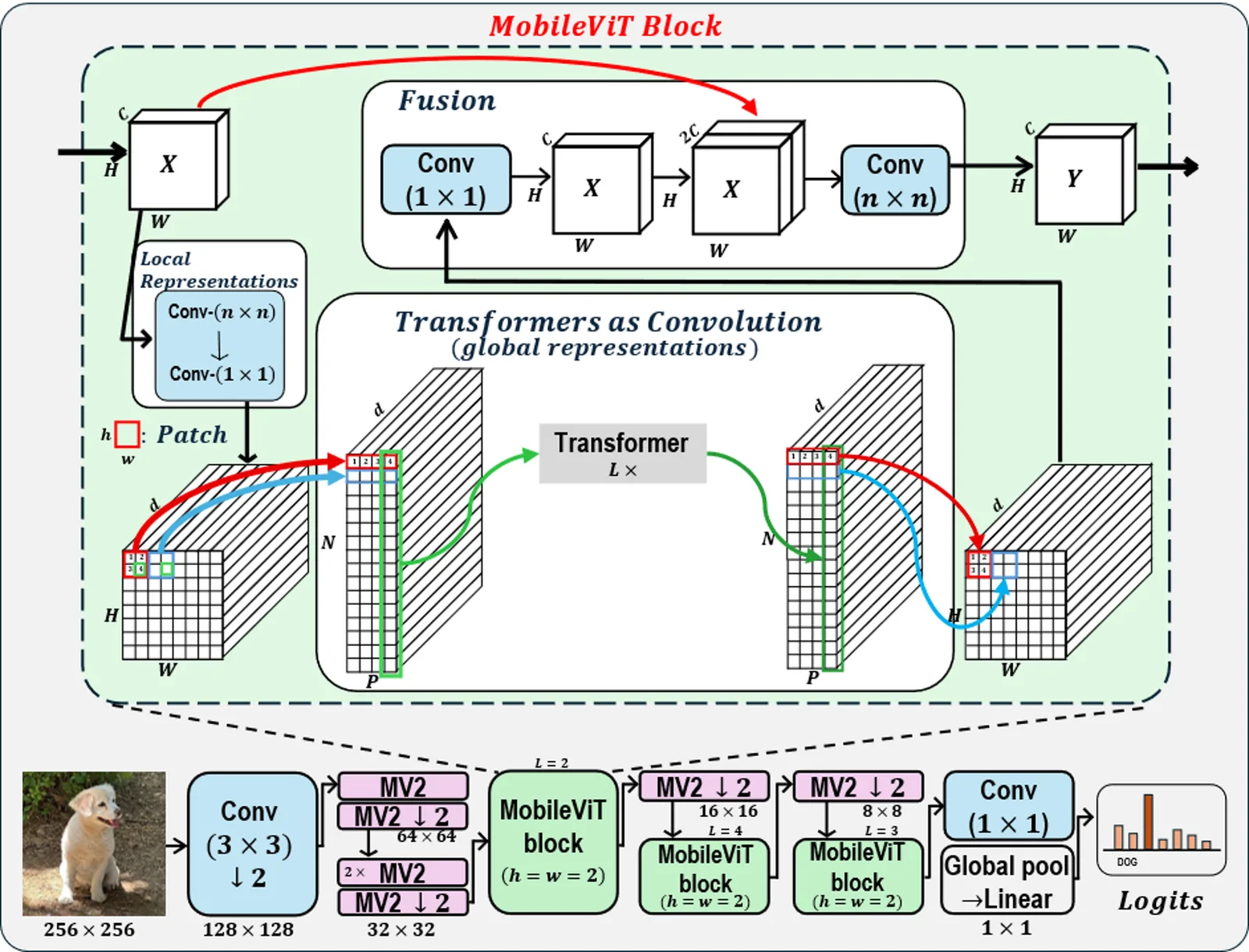
MobileViT architecture and MobileViT block [10]. MobileViT combines lightweight convolutional blocks with Transformer-based global representation learning. The MobileViT block first extracts local features with convolutions, unfolds them into patch representations for Transformer processing, and then folds the features back for convolutional fusion and classification

**MobileViT** was proposed as a lightweight and mobile-friendly vision Transformer that combines convolutional and Transformer-based representations [10]. The motivation was that lightweight CNNs are efficient and easy to deploy but primarily model local spatial relationships, whereas ViTs can model global interactions but are often heavier and less suitable for mobile devices. MobileViT addresses this by using convolutional layers for local feature extraction and Transformer layers for global representation learning. In a MobileViT block, feature maps are processed by convolutions, unfolded into patches, passed through Transformer layers, and then folded back and fused with convolutional features (Fig. 4) [10].

MobileViT is important in the development of image classification models because it represents an early effort to adapt Transformer-based visual representation to mobile-scale model budgets. Rather than applying a large vanilla ViT directly to mobile inference, MobileViT modifies the architecture to preserve CNN-like spatial inductive bias while incorporating global self-attention. The original work reported competitive ImageNet classification performance with small parameter counts and also evaluated latency on mobile hardware, emphasizing that practical efficiency depends on platform characteristics rather than FLOPs alone [10]. In this sense, MobileViT plays a role for Transformer-based image classification similar to MobileNet in the CNN lineage: it illustrates how a high-capacity model family can be redesigned for edge-oriented visual recognition .

## Our optimization of image classification model

### Token merging

Token Merging (ToMe) reduces ViT computation by gradually merging similar tokens inside existing Transformer blocks rather than directly deleting tokens [11]. Compared with token pruning, which suffers from inherent information loss, merging attempts to preserve part of the removed information by aggregating selected tokens.

**Fig. 5:**
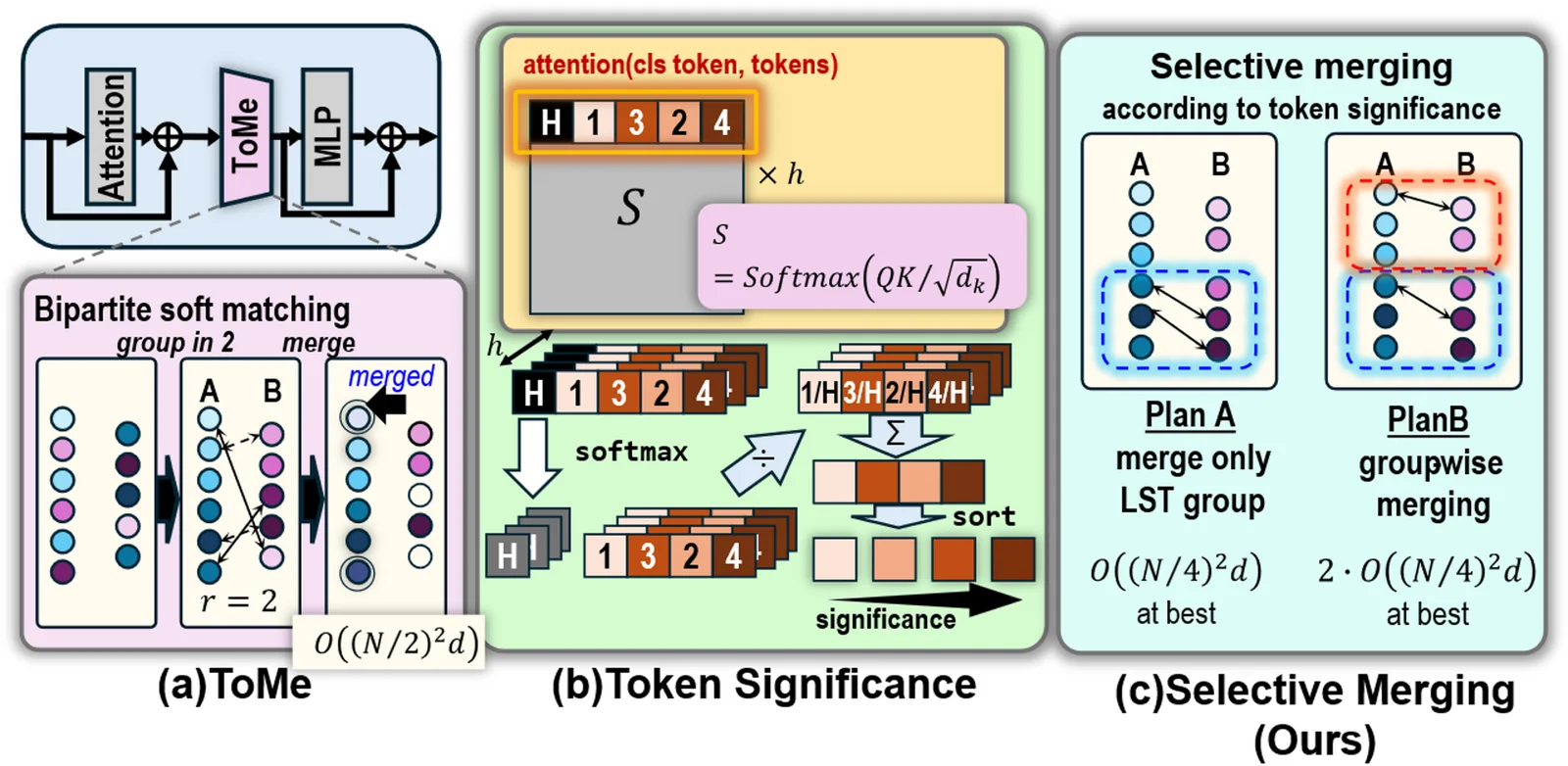
Token merging and significance-guided selective merging. **a** Standard Token Merging applies bipartite soft matching to merge similar tokens within the Transformer block. **b** Token significance is estimated from class-token attention, following the importance concept used in ViT-ToGo. **c** The proposed selective merging strategy applies ToMe according to token significance, either by merging only the LST group or by applying group-wise merging to MST and LST groups

The main cost in ToMe is determining which tokens should be merged. ToMe avoids exhaustive all-pairs token matching by partitioning tokens into two sets and applying bipartite soft matching based on token similarity, which is shown in Fig. 5a. Although this reduces the computational complexity by roughly a factor of 4 relative to full pairwise matching, the similarity matrix multiplication remains costly for edge device inference.

### Token significance

As prior works have shown, not all image tokens contribute equally to classification [7, 12]. Inspired by [13], token significance can be estimated from the class token attention distribution instead of evaluating full token-to-token importance independently.

ViT-ToGo [13] uses the first row of the MHSA attention probabilities, corresponding to the class token, as token significance indicators for image tokens while excluding the class token''s self-attention entry. It also accounts for the unequal contribution of different attention heads by using the class token''s self-attention probability in each head as a head importance estimate, where smaller values are treated as indicating more informative heads. Although [13] applies this estimate to grouped token pruning, we use the same attention-derived importance concept to identify low-importance tokens, which we refer to as *less significant tokens*, as summarized in Fig. 5b.

**Fig. 6:**
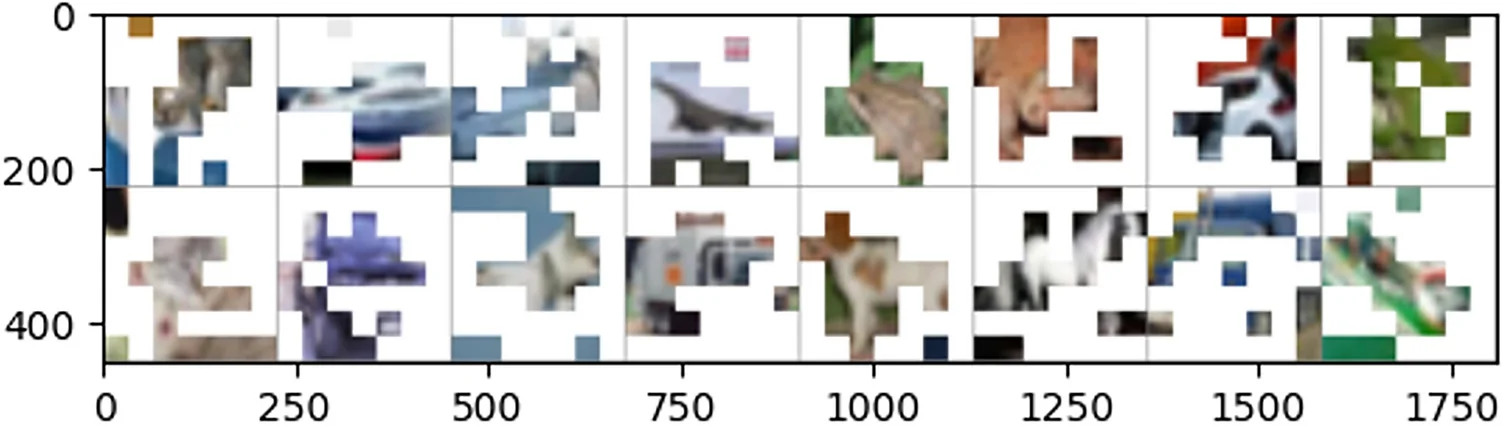
Visualization of token significance in ViT-B on CIFAR-10. Less significant tokens are pruned using a ViT-ToGo-inspired importance criterion with a pruning ratio of 0.2 at layers 4, 7, 9, and 11 [13]

Figure 6 qualitatively visualizes token significance by applying a ViT-ToGo-inspired token pruning criterion to ViT-B on CIFAR-10. Less significant tokens are pruned with a pruning ratio of 0.2 at layers 4, 7, 9, and 11 [13]. The resulting visualizations preserve most object regions while removing many background-dominant patches, supporting the use of attention-derived token significance for identifying less significant tokens.

### Proposed method: selective token merging

We propose a selective merging strategy, where token merging is applied according to token significance rather than over the entire token sequence. As illustrated in Fig. 5c, we consider two selective merging strategies. The first strategy, denoted as Plan-A, applies ToMe only to the least significant tokens (LSTs), leaving the most significant tokens (MSTs) unchanged. This is intended to merge tokens that show weaker contribution to the classification decision, thereby reducing accuracy degradation while preserving the throughput benefit of token reduction. The second strategy, denoted as Plan-B, applies ToMe separately to the MST and LST groups. This *group-wise strategy* is considered because forcing all merging to happen only within the LST group may produce suboptimal token pairs when the LSTs are not mutually similar (Fig. 6).

**Fig. 7:**
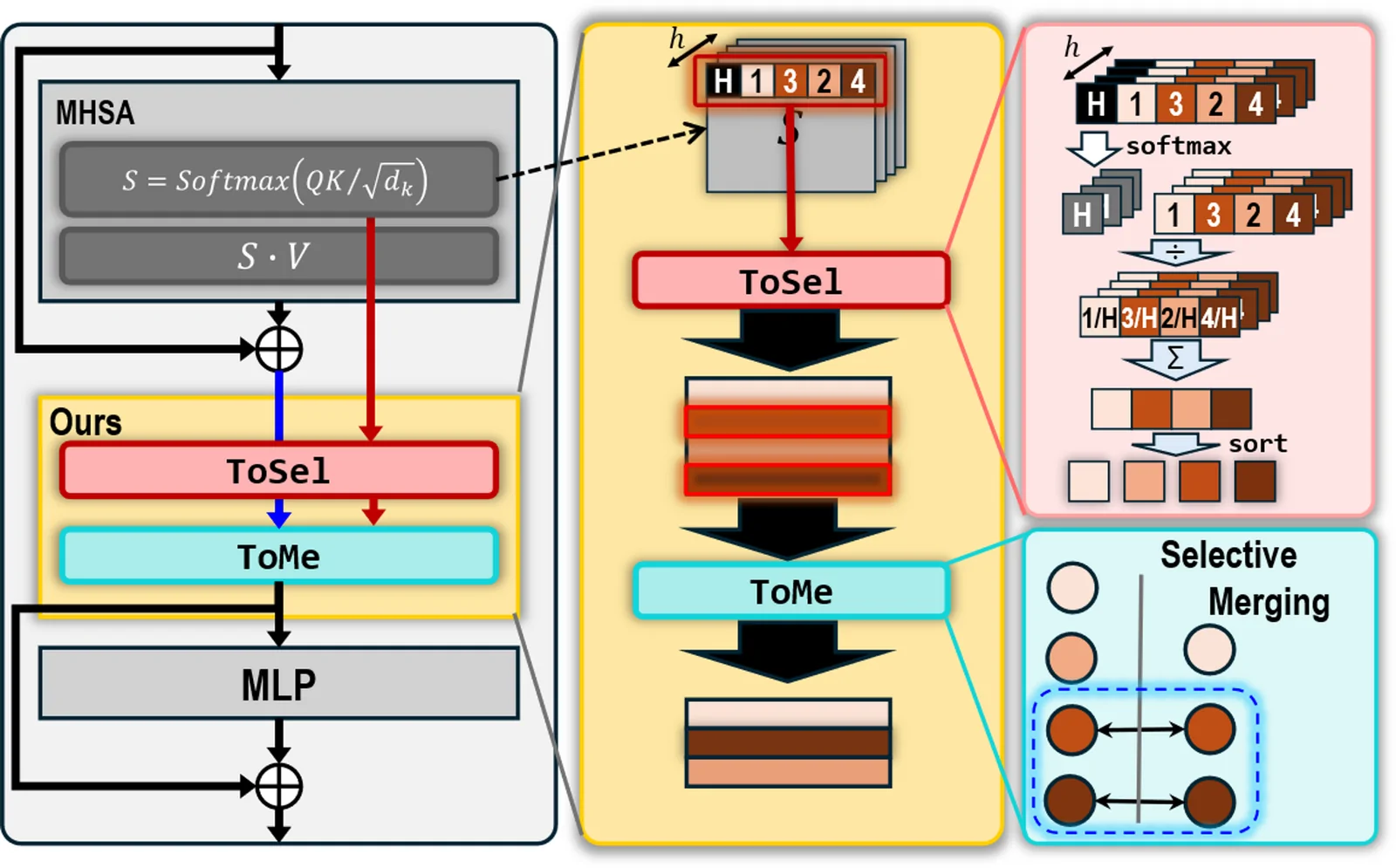
Proposed ToSel-assisted selective token merging architecture. The Token Selector reuses attention information from multi-head self-attention to identify least significant tokens and applies ToMe selectively before the MLP block. This structure reduces token-merging overhead while preserving tokens with higher classification significance

Figure 7 illustrates the Transformer block architecture with our proposed method applied. During MHSA, the attention path computes $Q{K}^{\top }$, applies softmax to obtain attention probabilities, and then performs the attention-value multiplication. We reuse these attention probabilities to compute token importance using a Token Selector (ToSel), which identifies the least significant tokens. After ToSel determines the merging candidates, ToMe is applied to the selected token group. ToSel adds ranking overhead because token importance is aggregated across attention heads and the tokens are ranked. With ToSel implemented by sorting the token-importance scores, this overhead is approximately $O\left(hN+N\mathrm{log}N\right)$. Restricting ToMe to a smaller token group reduces the matching workload because the token matching stage scales quadratically with the number of candidate tokens. For example, if the tokens are evenly divided into LST and MST groups, Plan-A in Fig. 5c reduces the bipartite matching matrix from $O\left({\left(N/2\right)}^{2}d\right)$ in standard ToMe to $O\left({\left(N/4\right)}^{2}d\right)$. Plan-B in Fig. 5c reduces the matching cost to $\left(2\cdot O\left({\left(N/4\right)}^{2}d\right)\right)$. Therefore, ToSel adds a ranking cost, but selective merging reduces the dominant quadratic matching cost by limiting ToMe to a smaller candidate set.

Overall, our selective merging strategy reduces the matching overhead and better preserves classification-relevant tokens, thereby improving the accuracy-throughput tradeoff over standard ToMe.

## Experiments

### Experimental setups

We evaluate the proposed selective token merging method on CIFAR-10 using ViT-Tiny as the baseline model. The ViT-Tiny model is fine-tuned with the timm library, and all inference experiments are conducted on a resource-constrained CPU platform to emulate an edge AI deployment environment. The hardware platform uses an Intel Core i5-1035G7 CPU at 1.20 GHz, with 7 GB available memory out of 16 GB total system memory and 3200 MT/s memory speed. The baseline and comparison methods are summarized in Table 1.

**Table 1:** Model and token-reduction setup for ViT-Tiny experiments

	ViT-Base	ViT-Tiny	ViT-Tiny ToMe	ViT-Tiny ToGo
Params	86 M	5.5 M	5.5 M	5.5 M
Patch size	16×16	16×16	16×16	16×16
# heads	12	3	3	3
Embed dim	768	192	192	192
MLP dim	3072	768	768	768
Layers	12	12	12	12
Merge/prune rate (per layer)	–	–	**13 tokens**	**25%**
Layer index	–	–	**All**	[4, 7, 8, 14]
Initial tokens	–	–	**196**	**196**
Final tokens	–	–	**40**	**62**

The table summarizes the architectural parameters of ViT-Base and ViT-Tiny, together with the merge/prune settings used for the ToMe and ToGo-inspired comparison methods. Bold data values indicate the token-reduction settings and resulting token counts.

For the additional experiments, we evaluated the timm implementations [15] of ViT-Tiny, ViT-Small, and ViT-Base on ImageNet-1 K. The three models contain 12 Transformer layers and use embedding dimensions of 192, 384, and 768 and 3, 6, and 12 attention heads, respectively. Top-1 accuracy was evaluated on ImageNet-1 K validation set [16, 17]. Throughput was measured at batch size 128. All additional experiments used the same local system, equipped with an Intel Core Ultra 7 265 K processor with a 3.9 GHz P-core base frequency and 32 GiB of dual-channel DDR5-5600 memory. Images from both CIFAR-10 and ImageNet-1 K were processed at an input resolution of *224× 224*.

The baseline is the fine-tuned ViT-Tiny model without token reduction. Standard ToMe is used as the token-merging reference, where token merging is applied uniformly across layers. The ViT-ToGo-inspired baseline applies token pruning according to attention-derived token importance, providing a pruning-based comparison to token merging. Our proposed methods add ToSel before ToMe and apply merging selectively according to token significance, using Plan A and Plan B to evaluate LST-only merging and group-wise merging, respectively.

### Experimental results and discussion

Table 2 reports the classification accuracy (%) and inference throughput (images/s) of the evaluated methods. The parameter *r* denotes the merge rate, where *r* tokens are merged at each applied layer. In the ToSel column, *[ratio]* denotes the ratio of least significant tokens (LSTs), and *layers* denotes the number of layers applied with that ratio, starting from the 0th layer.

**Table 2:** Experimental results on CIFAR-10 using ViT-Tiny

Merge rate (*r*)	ToSel ([*ratio*]*layers)	Model	Accuracy (%)	Throughput (images/s)
		Baseline (ViT-Ti)	**97.64 (‒0.00%)**	13.32 (1.00×)
		ToGo	92.65 (‒4.99%)	15.43 (1.16×)
		ToMe (*r*=13)	97.22 (‒0.42%)	**18.97** (1.42×)
13	[0.3]*12	ToSel+ToMe (Plan B)	97.29 (‒0.35%)	
	[0.5]*12	ToSel+ToMe (Plan B)	97.25 (‒0.39%)	
	[0.5]*4+[0.3]*2+[0.5]*6	ToSel+ToMe (Plan B)	**97.35 (‒0.29%)**	**17.67**(1.33×)
	[0.3]*12	ToSel+ToMe (Plan A)	97.27 (‒0.37%)	19.08 (1.43×)
	[0.3]*6+[0.0]*6	ToSel+ToMe (Plan A)	**97.41 (‒0.23%)**	19.04 (1.43×)
	[0.3]*6+[0.2]*6	ToSel+ToMe (Plan A)	97.37 (‒0.27%)	**19.29** (1.45×)
11	[0.5]*12	ToSel+ToMe (Plan B)	**97.37 (‒0.27%)**	**18.73** (1.41×)

Accuracy and CPU inference throughput are compared for the baseline ViT-Tiny, ToGo-inspired pruning, standard ToMe, and the proposed ToSel-assisted selective merging methods. The ToSel column denotes the LST-ratio schedule applied across Transformer layers. Bold data values indicate the highest accuracy or throughput within each comparison group.

**Plan A versus Plan B.** Plan-A applies ToMe only to the LST group, whereas Plan-B applies ToMe separately to the MST and LST groups. Both strategies preserve accuracy better than standard ToMe, but Plan-A shows the stronger accuracy-throughput tradeoff. In particular, Plan A with $\left[0.3\right]*6+\left[0.0\right]*6$ achieves 97.41% accuracy and 19.04 images/s, improving over standard ToMe with 97.22% accuracy and 18.97 images/s. This result indicates that merging only the LST group, which contains tokens that show weaker classification contribution, is more effective than group-wise merging.

**Ratio.** In Plan-B, the scheduled ratio $\left[0.5\right]*4+\left[0.3\right]*2+\left[0.5\right]*6$ improves accuracy over the uniform $\left[0.3\right]*12$ and $\left[0.5\right]*12$ cases. In Plan-A, applying selective merging mainly in shallow layers, as in $\left[0.3\right]*6+\left[0.0\right]*6$ and $\left[0.3\right]*6+\left[0.2\right]*6$, improves the accuracy-throughput tradeoff compared with the uniform [0.3]*12 setting. These results indicate that the optimal LST ratio varies across layers.

**Merge Rate.** Reducing the merge rate from *r=13* to *r=11* still provides throughput comparable to standard ToMe at *r=13* while improving accuracy. With *r=11*, the Plan-B setting reaches 18.73 images/s and 97.37% accuracy, compared with 18.97 images/s and 97.22% accuracy for standard ToMe at *r=13*. This result shows that selective merging can recover accuracy while maintaining most of the throughput gain from token merging.

### Layer scheduling and imageNet-1 K evaluation

We calibrated the selective-merging window using a 20,000-image, class-stratified subset sampled from the ImageNet-1 K training set, with 20 images per class. We fixed the LST selection ratio at 0.3 and the merge count at 13 in each layer where merging was applied. Selective merging was applied to six consecutive layers, and the window was shifted from layer indices 0–5 through 6–11 while all other settings remained unchanged. We selected the earliest window that improved Top-1 accuracy over standard ToMe across all three model variants while retaining a throughput improvement over the corresponding unmerged baselines. The 0–5 window remained 0.225–0.695 percentage points below standard ToMe, whereas the 1–6 window improved accuracy by 0.395–1.650 percentage points and retainedspeedups from *1.111× * to *1.343× *. We therefore selected layer indices 1–6 as the operating point for validation (Table 3).

**Table 3:** ImageNet-1K validation results for the selected ToMe+ToSel window

Model	Configuration	Token-merging layer indices	Accuracy (%)	Throughput (img/s)	Speedup
ViT-Tiny	Baseline	None	75.456 (0.000)	167.334	1.000×
	ToMe	0–11	73.010 (− 2.446)	250.867	1.499×
	**ToMe+ToSel (Ours)**	**1–6**	**74.256 (− 1.200)**	**178.838**	**1.069×**
ViT-Small	Baseline	None	81.386 (0.000)	57.446	1.000×
	ToMe	0–11	79.298 (− 2.088)	93.536	1.628×
	**ToMe+ToSel (Ours)**	**1–6**	**80.520 (− 0.866)**	**71.796**	**1.250×**
ViT-Base	Baseline	None	85.102 (0.000)	17.252	1.000×
	ToMe	0–11	83.738 (− 1.364)	29.723	1.723×
	**ToMe+ToSel (Ours)**	**1–6**	**84.518 (− 0.584)**	**23.268**	**1.349×**

Parentheses report percentage-point Top-1 changes from baseline; speedup is relative to each model’s baseline. Bold data values indicate the selected ToMe+ToSel configuration and its results.

**Fig. 8:**
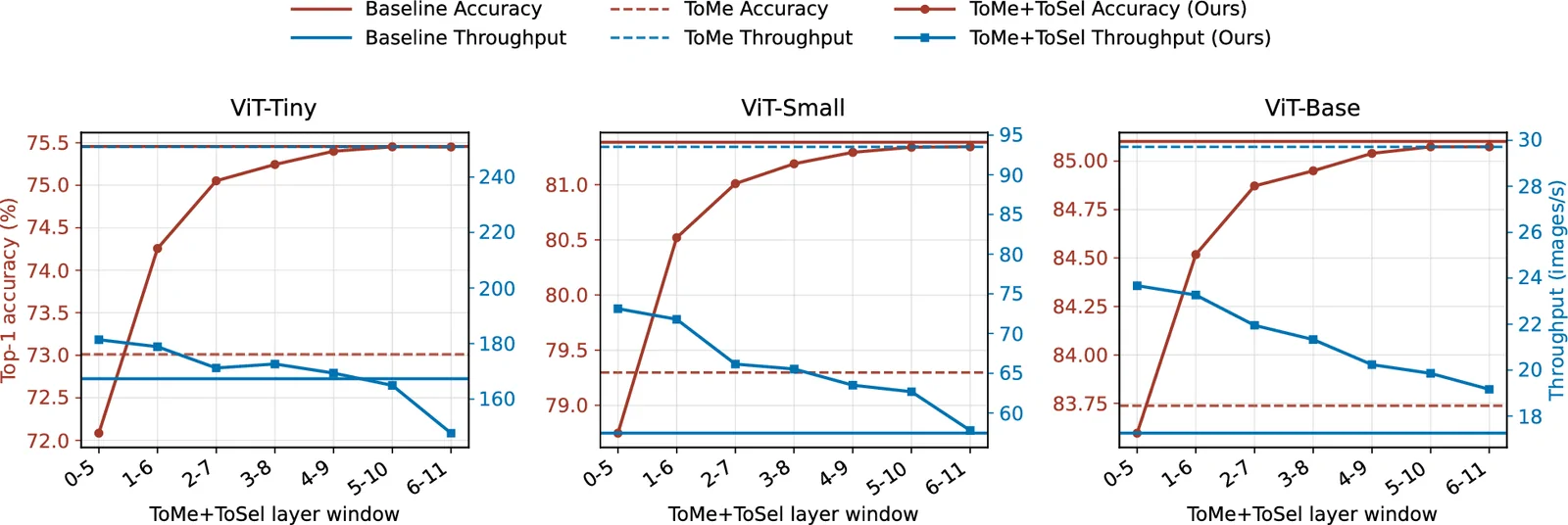
ImageNet-1K accuracy and throughput across six-layer ToMe+ToSel windows. Validation sweep across zero-based six-layer windows; baseline and standard ToMe are reference lines

We further performed the complete validation set sweep shown in Fig. 8 to examine whether the layer-dependent accuracy-throughput tendency persisted. Across ViT-Tiny, ViT-Small, and ViT-Base, moving the ToMe+ToSel window toward deeper layers better preserves Top-1 accuracy, which is consistent with deeper layers containing more refined, class-relevant token representations. However, merging on deeper layers reduces throughput because fewer subsequent Transformer layers benefit from the shortened token sequence. The selected window at layer indices 1–6 therefore provides a practical compromise between accuracy preservation and inference speedup.

Compared with standard ToMe, the selected ToMe+ToSel configuration improves Top-1 accuracy by 1.246, 1.222, and 0.780 percentage points for ViT-Tiny, ViT-Small, and ViT-Base, respectively. It retains speedups of *1.069× *, *1.250× *, and *1.349× * over the corresponding unmerged baselines. These results show that selective merging in the selected shallow-layer window preserves more of the baseline accuracy than standard ToMe while retaining a meaningful inference speedup. The consistent trend across ImageNet-1 K and all three ViT variants indicates that the proposed method generalizes across a larger dataset and multiple model capacities .

**Table 4:** Batch-1 ToSel-related latency on ImageNet-1K

Model	Model-forward latency (ms)	ToSel-related latency (ms)	Share of ToSel latency (%)
ViT-Tiny	19.432	0.788	4.057
ViT-Small	33.327	1.399	4.198
ViT-Base	73.159	1.798	2.458

### Quantitative ToSel-related latency

To quantify the complete ToSel-related overhead, we first captured the tensors required by ToSel during a normal model-forward pass. We then used the captured tensors to measure ToSel and gather-based token grouping in a separate isolated run. The measurement includes token-significance calculation, sorting, LST/MST index generation, and gather-based group construction. We used batch size 1 with an input from the ImageNet-1 K validation set and applied ToSel to layer indices 1–6 under zero-based indexing. The hardware configuration was the same as that used for the ImageNet-1 K throughput experiment. The median per-layer ToSel latencies from the six layers in which ToSel was applied were summed and expressed as a percentage of the separately measured median model-forward latency.

For ViT-Tiny, ViT-Small, and ViT-Base, the aggregated ToSel-related latencies are 0.788, 1.399, and 1.798 ms, corresponding to 4.057%, 4.198%, and 2.458% of median batch-1 model-forward latency, respectively. Thus, selection and token grouping remain a small fraction of inference latency across the three model variants (Table 4).

## Conclusion

This paper reviewed image classification models from CNN-based architectures to ViT-based architectures and proposed a selective token merging method for edge AI inference. The proposed method uses ToSel to identify least significant tokens and applies ToMe selectively, preserving the vanilla ViT backbone while reducing token-processing overhead. Experiments on CIFAR-10 with ViT-Tiny show that selective merging improves the accuracy-throughput tradeoff over standard ToMe. Additional ImageNet-1 K experiments with ViT-Tiny, ViT-Small, and ViT-Base show that selective-merging windows on deeper layers preserve accuracy more effectively, whereas windows of shallow layers provide greater throughput by reducing the token sequence for more subsequent layers. The selected shallow-layer window preserves more of the baseline accuracy than standard ToMe while retaining speedups across all three model variants. These results indicate that significance-guided selective merging generalizes across a larger dataset and multiple model capacities.

## Data Availability

The public dataset and external software resources used in this study are available from their original sources. The CIFAR-10 dataset is available at https://www.cs.toronto.edu/~kriz/cifar.html. The ImageNet-1 K (ILSVRC2012) dataset used in this study is available from the official ImageNet website, subject to registration and its terms of access: https://image-net.org/challenges/LSVRC/2012/2012-downloads.php. The timm library is available at https://github.com/huggingface/pytorch-image-models. The Token Merging implementation used as the basis for ToMe is available at https://github.com/facebookresearch/tome. The custom implementation, experimental configurations, and additional data generated during the current study are available from the corresponding author on reasonable request.
